# Characterizing chronic obstructive pulmonary disease using quantitative MRI biomarkers

**DOI:** 10.1093/bjr/tqaf249

**Published:** 2025-10-10

**Authors:** Daniel Genkin, Kalysta Makimoto, Miranda Kirby

**Affiliations:** Department of Physics, Toronto Metropolitan University, Toronto, ON M5B 2K3, Canada; Department of Physics, Toronto Metropolitan University, Toronto, ON M5B 2K3, Canada; Department of Physics, Toronto Metropolitan University, Toronto, ON M5B 2K3, Canada

**Keywords:** MRI, lungs, COPD, quantitative, imaging, biomarkers

## Abstract

Chronic obstructive pulmonary disease (COPD) is a heterogeneous lung disease that occurs due to structural changes to the parenchyma, airways and pulmonary vasculature, and consequent functional impairments to ventilation and perfusion. Although computed tomography (CT) imaging is the standard for assessing structural lung changes in COPD, it requires ionizing radiation and is unable to provide functional information without contrast agents. Conversely, there have been numerous developments for MRI of the lungs in the last several decades, allowing for the quantification of structural and functional abnormalities without ionizing radiation. Various quantitative MR (qMR) imaging biomarkers have been developed that describe parenchymal and airway structure as well as ventilation and perfusion within the lungs. These qMR imaging biomarkers have been investigated in individuals with COPD, reporting both cross-sectional and longitudinal associations with important outcomes. Therefore, the aim of this article is to briefly review some commonly used MRI techniques that have been investigated for lung imaging and discuss commonly implemented qMR imaging biomarkers and their application in COPD. Additionally, this review will focus on gaps in the literature that should be addressed to allow for future widespread implementation of qMR imaging biomarkers in COPD-related research.

## Introduction

Chronic obstructive pulmonary disease (COPD) is a leading cause of mortality worldwide.^1^ There is currently no known cure for COPD, and therefore management strategies aim to reduce symptoms, improve quality-of-life, and slow disease progression.^2^ According to Global Initiative for Chronic Obstructive Lung Disease (GOLD) criteria,^2^ a COPD diagnosis should be confirmed through a post-bronchodilator forced expiratory volume in 1 second (FEV_1_) to forced vital capacity (FVC) ratio (FEV_1_/FVC) < 0.70, measured by spirometry. However, spirometry is a single global measure of overall pulmonary function, and is not able to characterize the varying pathophysiologies that occur in individuals with COPD. Methods more sensitive to the structural/functional changes that occur in COPD are required, such as lung imaging modalities.

In COPD, chest computed tomography (CT) imaging is the gold standard to assess the extent of emphysema^3^ and abnormal airway remodelling.^4^ Advances in image analysis algorithms allow for automated extraction of a wide variety of quantitative CT (qCT) biomarkers that characterize the disease within the lung parenchyma, airways, and vasculature. Accordingly, there is a large and growing body of literature of observational studies demonstrating associations between qCT biomarkers with histopathology,^5^ and various COPD outcomes.^6^^,^^7^ Nonetheless, the ionizing radiation concerns of CT imaging, particularly when longitudinal monitoring is required, motivates the use of other imaging modalities that do not suffer from the same drawback, such as MRI.

Lung MRI has traditionally suffered from difficulties due to 3 major concerns: (1) low proton density; (2) fast signal decay; and (3) susceptibility to motion artefacts.^8^ Recent advances in MRI techniques have uniquely overcome these limitations. For example, the administration of exogenous paramagnetic materials like gadolinium (Gd), fluorine (^19^F), and hyperpolarized gases like xenon (^129^Xe), or helium (^3^He), enables direct quantification of ventilation and perfusion in the lungs.^9^ Alternatively, proton-based (^1^H) ultra-short echo time (UTE) MRI pulse sequences allow for the sampling of lung tissue data before complete loss of signal occurs, providing up to submillimetre visualization of the parenchyma and airways.^10^ Lastly, the introduction of respiratory-gating (breath tracking) during scanning, along with Fourier decomposition-based analysis algorithms such as phase-resolved functional lung (PREFUL), permit the voxel-wise discrimination of both the respiratory and cardiac cycles using ^1^H-MRI, thereby discerning ventilation and perfusion-related information.^9^^,^^10^ However, given the novel nature of these advancements, application of these MRI techniques and their derived quantitative MR (qMR) imaging biomarkers in COPD is still emerging. Therefore, the purpose of this review is to: (1) offer succinct descriptions of each structural and functional lung MRI technique; (2) provide summaries of the most common qMR imaging biomarkers and current clinical evidence of their application for COPD; and (3) identify key gaps in the literature and areas of future research.

## Lung MRI techniques

**Figure 1.**
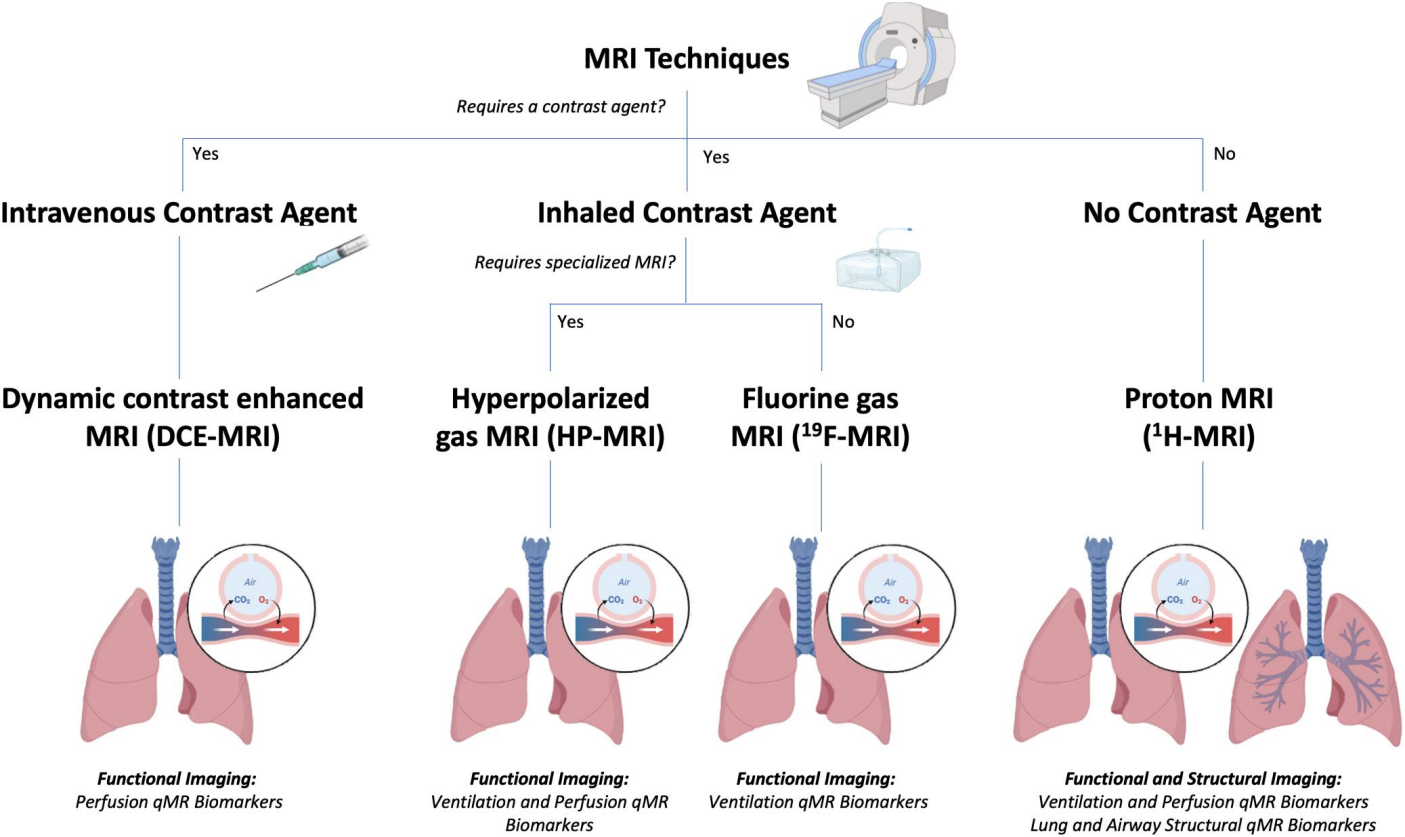
Flow chart demonstrating the feasibility of common lung MRI techniques dependent on available equipment to obtain functional or structural imaging. With intravenous contrast agents, functional imaging of perfusion can be acquired using dynamic contrast-enhanced MRI (DCE-MRI). With common inhaled contrast agents, functional imaging of ventilation can be acquired using fluorine gas MRI (^19^F-MRI). With access to specialized equipment such as a polarizer and less common inhaled contrast agents like hyperpolarized helium (^3^He) or xenon (^129^Xe), functional imaging of ventilation and gas-exchange can be acquired using hyperpolarized gas MRI (HP-MRI). Finally, proton MRI (^1^H-MRI) can be used to acquire CT-like structural images using ultrashort echo-time (UTE) pulse sequences, while also acquiring both ventilation and perfusion functional imaging during free-breathing using phase resolved functional lung (PREFUL) analysis. Figure created with www.Biorender.com.

Various MRI techniques are currently used for lung imaging including dynamic contrast-enhanced MRI (DCE-MRI), hyperpolarized-gas MRI (HP-MRI), fluorine-gas MRI (^19^F-MRI), and proton MRI (^1^H-MRI). Figure 1 illustrates a flow chart for determining which MRI technique can be implemented depending on equipment availability and type of information to be extracted. Table 1 reports the advantages and disadvantages of each MRI technique. Although other MRI techniques like oxygen-enhanced MRI and arterial spin labelling MRI have been investigated for lung imaging, the focus of this review is strictly on the 5 aforementioned techniques, as they are the most commonly implemented in literature for COPD-related research.

**Table 1.** Summary of the advantages and limitations for each described lung MRI technique.

Lung MRI technique	Advantages	Limitations
Dynamic contrast-enhanced MRI	Can directly visualize lung function (perfusion).	Requires an intravenous contrast agent which has potential side effects.
Hyperpolarized-gas MRI	Can directly visualize lung function (ventilation) Can probe the gas-exchange process directly.	Requires inhaled gas. Requires specialized MR equipment (eg polarizer, coils).
Fluorine-gas MRI	Can directly visualize lung function (ventilation).	Requires inhaled gas. Requires specific hardware (eg broadband multinuclear amplifier).
Proton MRI	Can directly visualize lung structure and indirectly visualize lung function (perfusion and ventilation). Does not require any contrast agents (intravenous contrast agent or inhaled gases). Does not have specific hardware requirements.	Dependent on the coils used for image acquisition. Produces images with arbitrary units.

### Dynamic contrast-enhanced MRI

DCE-MRI is a functional imaging technique that utilizes a contrast agent to visualize perfusion in the lungs. Intravenous injections typically deliver the contrast agent directly to the arterial/venous system over a few minutes, allowing for immediate absorption.^11^ Common contrast agents include manganese chelates, iron oxide particles, or the most commonly used, gadolinium chelates.^12^ DCE-MRI scans are typically acquired during breath-hold,^12^ but recent work has shown the ability to collect scans during free-breathing.^13^ It has also been demonstrated that DCE-MRI scans offer comparable visualization of the intrapulmonary vascular perfusion compared to gold-standard contrast-enhanced CT.^12^ Gadolinium-based contrast agents may cause mild side effects including nausea, rashes and/or hives, or more severe side effects including respiratory or cardiac arrest, or even systemic fibrosis and kidney failure in those with chronic lung disease, and more research is required to discern the dangers of repeated exposure.^11^^,^^14^

### Hyperpolarized-gas MRI

To mitigate potential side effects from intravenous contrast agents, hyperpolarized gases can be used as relatively safe and well-tolerated inhaled contrast agents.^15^ The most commonly used inhaled-contrast agent gases for HP-MRI include ^3^He and ^129^Xe.^16^ However, in recent years ^129^Xe gas is primarily used due to the relative shortage and greater financial burden of ^3^He.^17^ To generate an HP-MRI scan, the gas first needs to be hyperpolarized using a polarizer, and then delivered to the patient for inhalation using a dose bag.^16^ Once inhaled, the MRI scan is acquired, typically at breath-hold, but also potentially during free-breathing to visualize gas distribution (ie, ventilation) within the lungs. Notably, solely ^129^Xe-MRI is capable of directly imaging gas-exchange due to its solubility and unique resonance within different compartments of the lung, including the alveoli space, alveolar membrane, and the red blood cells. HP-MRI has been shown to provide equivalent ventilation information compared to single photon emission computed tomography (SPECT), the current clinical standard for functional imaging in patients with lung disease.^18^ However, due to the requirements for specialized equipment (polarizer, coils, etc), HP-MRI is more expensive than other lung MRI techniques and is not easily accessible.

### Fluorine-gas MRI

A more recently developed MRI technique that utilizes inhaled gas as a contrast agent, but does not require a polarizer, is ^19^F-MRI.^19^ 19F-MRI utilizes inert fluorinated gases, typically consisting of 79% perfluoropropane and 21% oxygen, that are inhaled and imaged during breath-hold or free-breathing to visualize ventilation within the lungs.^20^ Although ^19^F-MRI does not require a polarizer, it does have specific hardware requirements, including a broadband multinuclear amplifier.^20^ The delivery of the fluorinated gas mixture is done either manually, using large dose bags, or automatically, using computer-controlled delivery systems.^20^ However, comparison between ventilation information captured by ^19^F-MRI and SPECT has not been assessed. Furthermore, although ^19^F-MRI is more accessible and easier to implement than HP-MRI, it still requires inhaled-gas to act as a contrast agent.

### Proton MRI

Conventional proton MRI (^1^H-MRI) has historically been overlooked in applications related to lung disease as the low tissue density and quick signal decay have made it challenging to visualize the pulmonary structures.^9^^,^^10^ Advancements in ^1^H-MRI pulse sequences, in particular using ultrashort echo time (UTE) sequences with TEs < 2 ms, enable sampling of tissue signal before complete decay can occur.^10^ UTE-^1^H-MRI scans can be created with submillimetre spatial resolution and offer comparable visualization of the pulmonary structures as CT scans.^21^ Image collection for UTE-^1^H-MRI can occur at breath-holds of less than 20 s^22^ and during free-breathing with either retrospective or prospective respiratory-gating to correct for respiratory motion artefacts.^23^ Other motion correction techniques using navigator echoes and/or cardiac gating may also be used to track the positions of surrounding organs such as the diaphragm, chest wall, and heart to minimize the potential effect of cardiac and bulk motion. In addition to providing structural information, free-breathing ^1^H-MRI collects images during the complete respiratory and cardiac cycles. Registering all collected images to a common inflation state and subsequently performing a time-series voxel-wise analysis using the recently developed phase-resolved functional lung MRI (^1^H-PREFUL-MRI) technique sorts images according to their respiratory and cardiac cycle phase to visualize functional information related to ventilation and perfusion.^24^ It has been demonstrated that ^1^H-PREFUL-MRI obtains similar perfusion measurements as SPECT.^25^ Importantly, unlike the other imaging techniques, ^1^H-MRI related techniques do not require any exogenous paramagnetic materials or specific equipment to obtain images, and simply utilize specialized pulse sequences making them more accessible, less costly, and logistically challenging than methods that use contrast agents. However, UTE-^1^H-MRI images may be impacted by bias field and inhomogeneities that vary with scanner coils, and further consists of arbitrary units,^26^ which can potentially lead to challenges with standardization between datasets.

## Lung MRI biomarkers

### Structural biomarkers

#### Lung parenchyma visual scoring

**Figure 2.**
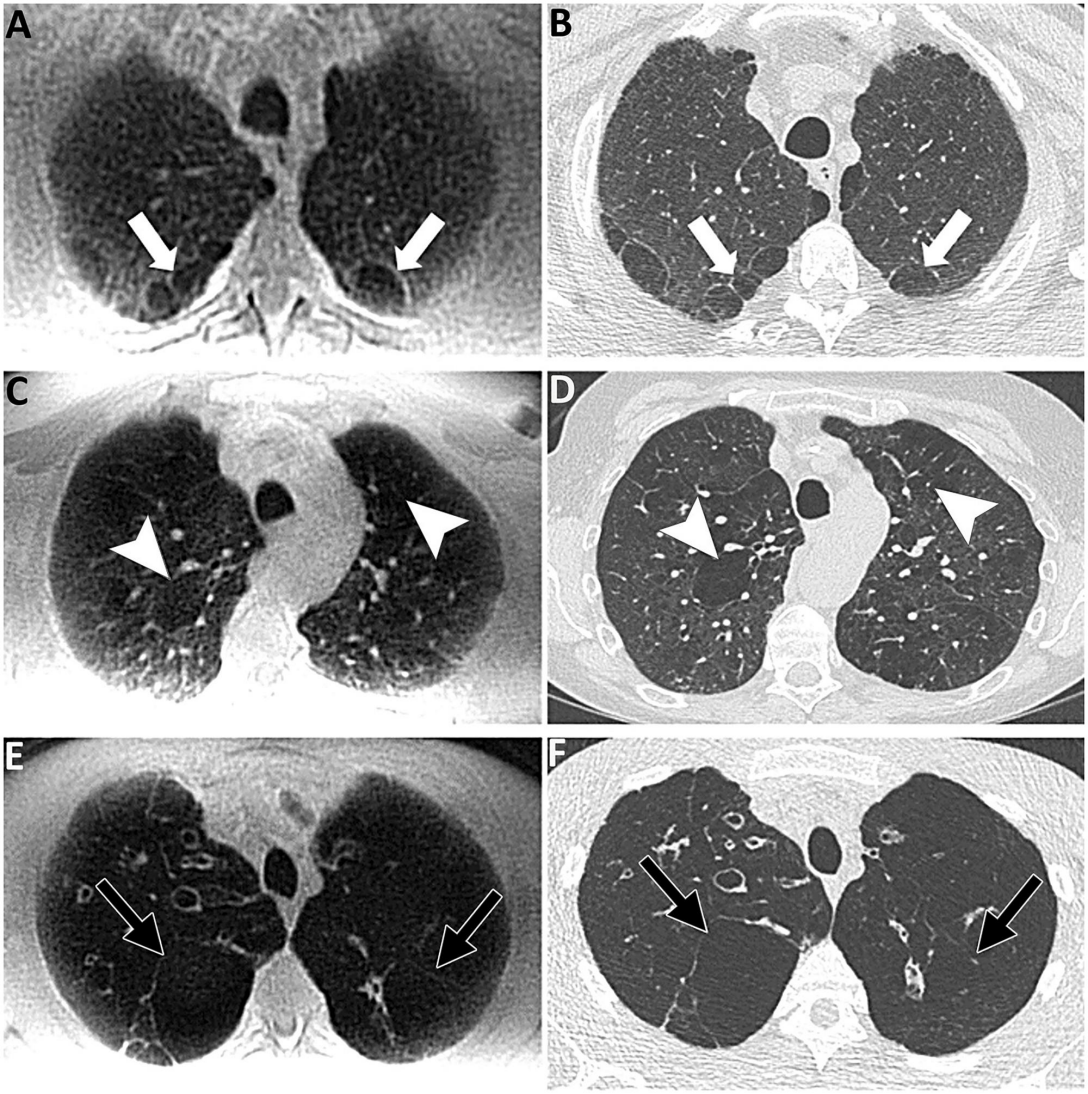
Three-dimensional images obtained at, A, C, E, ultrashort echo time MRI and, B, D, F, CT in 3 study participants with chronic obstructive pulmonary disease. A, B, Images in the first participant show mild paraseptal emphysema (arrows). C, D, Images in the second participant show moderate emphysema (arrowheads indicate areas of centrilobular emphysema). E, F, Images in the third participant show severe panlobular emphysema; there are conspicuous fine septal lines delineating hyperinflated lobules (arrows). Reproduced with permission from Benlala I, et al. Radiology, 2019, Jul;292(1):216-225. © 2019 Radiological Society of North America.^27^

Destruction of the alveolar tissue in individuals with COPD leads to persistent and frequently progressive airflow obstruction. Emphysema on chest CT imaging, typically taken at full inspiration (total lung capacity [TLC]) is visualized as decreased tissue density, with excellent contrast in comparison to healthy parenchyma.^2^ Recent literature using UTE-^1^H-MRI collected during free-breathing but reconstructed to functional residual capacity (FRC), has also shown that it is capable of detecting parenchymal abnormalities such as emphysema, visualized in Figure 2.^27^ Reconstruction of free-breathing images to FRC or collection during FRC breath-hold is preferred for UTE-^1^H-MRI to minimize the effect of respiratory motion artefacts. However, this may introduce potential differences in the visualization of emphysema as compared to the CT scans taken at TLC. Nevertheless, repeatability for visual scoring of emphysema in UTE-^1^H-MRI scans of adults with various lung diseases has yielded good results relative to low-dose CT scans (CT: ICC = 0.68; MRI: ICC = 0.61),^21^^,^^28^ including in those with COPD (CT: Kappa = 0.94; MRI: Kappa = 0.74).^29^ Similarly, good agreement between UTE-^1^H-MRI visual scoring with CT visual scoring of emphysema has also been reported (Kappa = 0.78).^21^^,^^29^

#### Lung parenchyma qMR biomarkers

Quantitative methods describing emphysema on UTE-^1^H-MRI scans have also been introduced. Segmentation of the lung parenchyma on UTE-^1^H-MRI can be performed using seeded-region growing,^30^ or deep learning-based segmentation methodologies, which are now the standard for lung segmentation.^31^ Research from multiple groups has shown feasibility of deep learning for UTE-^1^H-MRI lung segmentation with excellent performance relative to manual segmentation.^32–35^ However, only 2 of the aforementioned studies have released their models as open-source^33^^,^^35^ and evaluation of performance in individuals with COPD is limited. Furthermore, image acquisition protocols within UTE-^1^H-MRI scans themselves can vary (field strength, pulse sequence, breathing technique, etc), and the reproducibility of UTE-^1^H-MRI lung segmentation under changing acquisition protocols needs further investigation.

Variability in the definition of quantitative UTE-^1^H-MRI emphysema exists in literature. The current reported biomarkers include: the average signal intensity in the lung,^36^ the 15th percentile of signal intensity in the lung,^37^ or the percentage of lung tissue <10% of the mean thoracic soft-tissue signal intensity.^38^ Nevertheless, these studies have reported that UTE-^1^H-MRI emphysema measurements were significantly higher in individuals with COPD compared to controls, and were significantly correlated with gold standard CT-based emphysema measurements (low attenuation area ≤−950 Hounsfield Units [HU] [LAA_950_]) and lung function.^36–38^ More recently, a qMR biomarker known as low signal volume (LSV), defined as the proportion of the lung smaller than its 20th percentile signal intensity, was best correlated with LAA_950_ in comparison to other percentiles (*r* = −0.80; *P* < .001), and was significantly elevated in severe-COPD compared to mild-COPD patients.^27^ It is important to note that the LSV was quantified using UTE-^1^H-MRI scans taken at FRC, which may underestimate the amount of emphysema relative to scans taken at TLC as shown in CT studies,^39^^,^^40^ and further research is required to understand the impact of lung volume on qMR emphysema measurements. Furthermore, unlike in CT,^5^ there have been no studies that have histopathologically validated the ideal signal intensity threshold to define MRI-based emphysema.

#### Airway visual scoring

**Figure 3.**
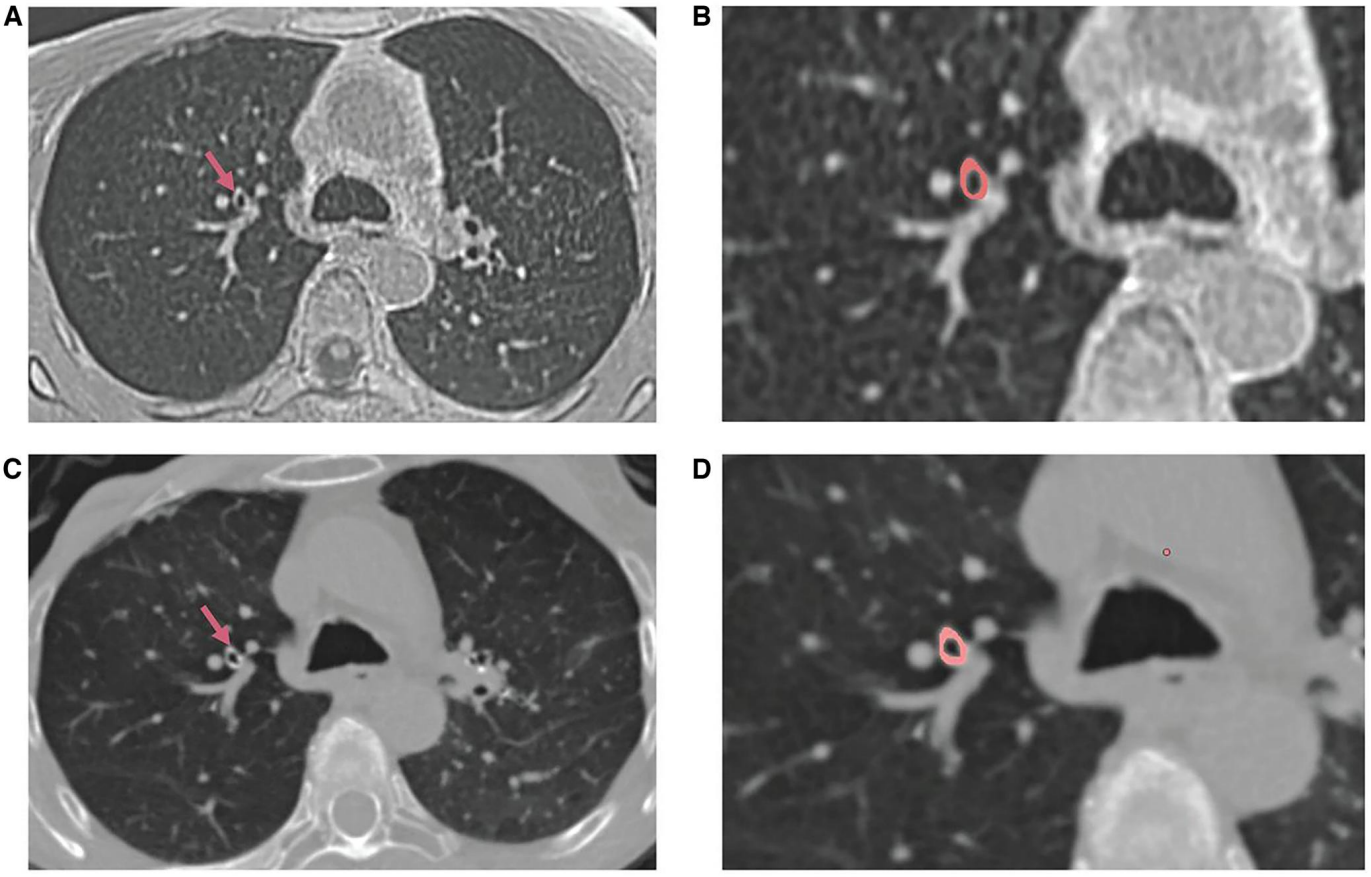
Representative lung images from a 59-year-old patient with severe asthma. Visualization of wall area measurements of RB1 (right upper lobe) (red arrow) using magnetic resonance imaging with ultrashort echo time pulse sequence (A, B) or computed tomography (C, D). Reproduced with permission of the ERS 2025 from Benlala I, et al. Eur Respir J, 2022; 59: 2100329. © 2022 European Respiratory Society.^46^

Airway abnormalities are a key morphological feature in individuals with COPD, and airway structure can be readily visualized on UTE-^1^H-MRI.^41^ Indeed, visibility of the airways up to the subsegmental generation by human observers has been reported on UTE-^1^H-MRI in healthy participants and those with various lung diseases like cystic fibrosis (CF) and interstitial lung disease (ILD).^28^^,^^42^^,^^43^ Furthermore, good-excellent repeatability for visual scoring of airway remodelling on UTE-^1^H-MRI is established,^21^^,^^44^ with one study showing similar repeatability to CT visual scoring in patients with CF.^45^ Moderate-high correlations between UTE-^1^H-MRI visual scoring and CT visual scoring for identifying both bronchiectasis and airway wall thickening have also been demonstrated in healthy participants and patients with CF.^44^Figure 3 highlights the ability of UTE-^1^H-MRI to visualize the airway wall for a segmental airway in the right upper lobe in a patient with severe asthma.^46^

**Figure 4.**
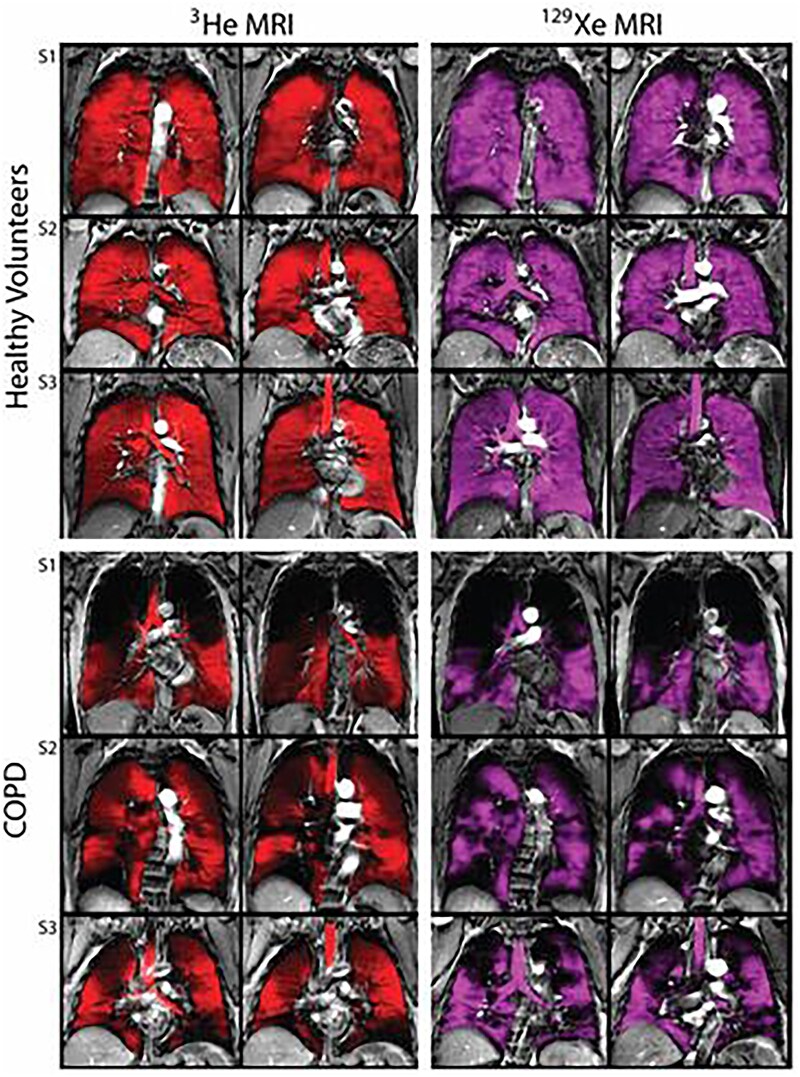
Static ventilation 3He and 129Xe MR images in 3 healthy volunteers and 3 patients with COPD. Images of the 2 coronal center sections, where trachea and 2 main bronchi are clearly visible, are registered to greyscale 1H MR images of thorax. Volunteer in S1 is 75-year-old woman with percentage predicted FEV1 of 93% and FEV1/FVC of 70%; volunteer in S2 is 57-year-old man with percentage predicted FEV1 of 95% and FEV1/FVC of 72%; volunteer in S3 is 51-year-old man with percentage predicted FEV1 of 120% and FEV1/FVC of 83%. Patient in S1 is 77-year-old woman with percentage predicted FEV1 of 50% and FEV1/FVC of 20%; patient in S2 is 68-year-old woman with percentage predicted FEV1 of 59% and FEV1/FVC of 53%; patient in S3 is 71-year-old man with percentage predicted FEV1 of 107% and FEV1/FVC of 58%. 3He gas distribution is displayed in red and 129Xe gas distribution is displayed in purple. Reproduced with permission from Kirby et al, Academic Radiology, 2012; 19(2):141-52. © 2012 Elsevier.^30^

#### Airway qMR biomarkers

UTE-^1^H-MRI airway tree segmentation, the proprietary step in extracting quantitative airway biomarkers, has shown promising recent progress. Semiautomatic airway segmentation from UTE ^1^H-MRI has been demonstrated for the trachea in neonates,^47^^,^^48^ and up to the segmental airways in paediatric and adult patients with CF and asthma.^49^^,^^50^ Good repeatability of UTE-^1^H-MRI airway tree segmentations in paediatric patients has also been reported.^49^ Alternatively, airway qMR imaging biomarkers extracted from these airway tree segmentations are still very much in the developmental phase, with ongoing efforts mostly focused on proof-of-concept studies. In neonates, changes in UTE-^1^H-MRI lumen area measurements of the trachea between inspiration and expiration offer a non-invasive diagnostic technique to quantify tracheomalacia.^47^^,^^48^ UTE-^1^H-MRI lumen diameter measurements of paediatric patients with CF and asthma have shown excellent repeatability for the trachea and main bronchi, but have been poor-moderate for the segmental airways.^49^ On the other hand in adults with asthma, segmental airway wall measurements taken using UTE-^1^H-MRI have shown moderate-good repeatability and good correlations with CT measurements,^46^ lending credibility for UTE-^1^H-MRI to quantitatively evaluate airway abnormalities at these airway generations in older patients. Furthermore, UTE-^1^H-MRI airway wall measurements have been demonstrated to be significantly elevated in CF and severe asthma patients relative to healthy and non-severe asthmatic controls.^46^^,^^50^

However, no studies have evaluated UTE-^1^H-MRI airway imaging biomarkers in COPD patients. Further, as fully automated airway segmentation down to the subsegmental airways is now possible in CT scans using open-source deep-learning models,^51^ future work should look to develop similar models for application in UTE-^1^H-MRI scans. This is especially important as synthetic generation of CT-like images from UTE-^1^H-MRI scans is now possible,^52^ and if reversed, may offer a pipeline to quickly generate the many ground-truths required for model training.

### Functional biomarkers

#### Ventilation biomarkers

Ventilation abnormalities in individuals with COPD occur due to prolonged lung emptying caused by airway narrowing or blockage, as well as loss of elastic recoil due to emphysema. The most commonly reported qMR imaging biomarker used to quantify ventilation abnormalities in COPD is the ventilation defect percent (VDP), or simply, the proportion of the lung that is poorly ventilated. Extracting VDP measurements requires dual structure-function MRI; ^1^H-MRI scans are first used to segment the lungs, and are then co-registered to functional ventilation maps. These maps are typically created using HP-MRI,^53–60^ but more recently, ^19^F-MRI61 and ^1^H-PREFUL-MRI24 have also shown the capability of creating ventilation maps. However, the lack of a clear lung border on HP-MRI and ^19^F-MRI images and potential for lung segmentation error on ^1^H-MRI images may lead to challenges in landmark alignment and by-extension accurate registration. To tackle this problem, complete automated pipelines performing lung segmentation and registration have been developed,^62–64^ but there is still widespread methodological heterogeneity between studies, which results in differences in VDP measurements.^65^ Alternatively, novel work has shown that it is possible to synthetically create HP-MRI ventilation maps directly using deep-learning with excellent overlap to ground-truths, bypassing the registration step altogether.^66^ Finally, the definition of a “ventilation defect,” the key step in transforming the ventilation maps into binarized defect masks, is still also subject to debate, with no current consensus in the literature^67^; a standardized approach for registration, segmentation, and defect identification is required.

#### HP-MRI ventilation applications in COPD

VDP quantified using HP-MRI has demonstrated moderate-high repeatability in patients with cystic fibrosis (CF),^67^^,^^68^ and reproducibility at different imaging sites in patients with asthma^69^; studies quantifying HP-MRI VDP repeatability and reproducibility in those with COPD are limited. In individuals with COPD, studies have demonstrated significant associations between HP-MRI VDP with important cross-sectional outcomes like FEV_1_ and FEV_1_/FVC measurements,^54^ symptom scores,^57^ and exercise capacity,^70^ and longitudinal outcomes like lung function decline,^57^ exacerbations,^53^ and mortality.^55^ The ability of HP-MRI VDP to quantify ventilation abnormalities is shown in Figure 4^71^; compared to healthy volunteers, individuals with COPD have ventilation defects throughout the lung. Further, multiple studies in ex-smokers and patients with asthma have shown associations between structural lung qCT biomarkers like total airway count (TAC),^56^ functional small airways disease (fSAD),^58^ and mucus plugs^59^ with HP-MRI VDP, and may offer a morpho-functional link between areas of poor ventilation and the manifestation of structural abnormalities. Importantly, it has been demonstrated that HP-MRI VDP can quantify subtle improvements in ventilation post-administration of inhaled long-acting beta-agonist/long-acting muscarinic receptor antagonist (LABA/LAMA) bronchodilators in those with moderate-severe COPD,^60^ however, more work is required to evaluate its role as a therapeutic endpoint for clinical trials.

#### ^19^F-MRI ventilation applications in COPD

VDP quantified using ^19^F-MRI ventilation maps has demonstrated strong correlations with HP-MRI VDP.^72^ Good repeatability for ^19^F-MRI VDP measurements has been demonstrated,^73^ but analysis was limited to healthy participants and reproducibility across various sites and scanner settings has not been assessed. ^19^F-MRI VDP has shown to be able to discriminate between healthy participants and those with COPD, but associations to important cross-sectional COPD outcomes have been limited to FEV_1_.^61^^,^^74^ Therefore, studies are required to determine associations with other important COPD outcomes, like symptoms and exercise capacity, as well as longitudinal outcomes. Similar to HP-MRI, ^19^F-MRI VDP has also been shown to be sensitive to ventilation changes following nebulized salbutamol-based bronchodilator administration,^74^ and the modality may also be suitable as a therapeutic endpoint, however more research is required.

#### 1H-PREFUL-MRI ventilation applications in COPD

The recently developed ^1^H-PREFUL-MRI analysis technique allows for generation of dual structure-function maps from a single free-breathing session, and by extension, simplifying the extraction of VDP to require only a single MR scan. VDP extracted from ^1^H-PREFUL-MRI has demonstrated strong correlations to both HP-MRI VDP^75–77^ and ^19^F-MRI VDP in those with COPD.^78^ Furthermore, good repeatability in those with COPD,^79^^,^^80^ and reproducibility at different field strengths for healthy volunteers^81^ have been established for ^1^H-PREFUL-MRI VDP, but the measurement has shown susceptibility to varying echo times in those with asthma and pulmonary fibrosis.^82^ Similarly to ^19^F-MRI VDP, ^1^H-PREFUL-MRI VDP discriminates between healthy participants and those with COPD,^76^^,^^78^ but associations to important cross-sectional COPD outcomes have also been limited to FEV_1_,^75^^,^^76^ and there is a need to investigate associations with longitudinal outcomes. Lastly, ^1^H-PREFUL-MRI VDP has also been shown to quantify subtle improvements in ventilation post-administration of indacaterol/glycopyrronium (IND/GLY) based bronchodilators in those with COPD.^83^

#### Additional ventilation biomarkers

Although VDP is the most commonly used qMR imaging biomarker to quantify ventilation abnormalities, additional ventilation biomarkers have been developed and implemented in COPD. Assessments of “raw” ventilation throughout the respiratory cycle in the lungs can be extracted through HP-MRI, ^19^F-MRI VDP, and ^1^H-PREFUL-MRI using quantitative biomarkers described as fractional, specific, or regional ventilation (FV, SV, and RV).^60^^,^^84^^,^^85^ FV measurements have shown strong correlations with lung function in individuals with COPD,^76^^,^^86^ but further investigations into other important cross-sectional and longitudinal COPD outcomes are limited, and should be the focus of future work.

#### Perfusion biomarkers

Consistent reductions in fresh oxygen uptake to the alveoli, also known as alveolar hypoxia, leads to coupled hypoxic pulmonary vasoconstriction in those with COPD, and consequently diminished pulmonary gas-exchange.^87^ Reduced pulmonary blood flow is thought to be a contributing mechanism for the worsening of pulmonary hypertension and eventual heart failure in COPD.^87^ Areas of hypoperfusion can be quantified using a qMR imaging biomarker known as perfusion defect percent (QDP), or the proportion of the lung that has poor gas-exchange. Similar to VDP, extracting QDP measurements requires dual structure-function MRI to bound functional gas-exchange maps created using DCE-MRI^88^ or ^1^H-PREFUL-MRI^89^ analysis, to the lungs. Methodological heterogeneity also exists for the definition of hypoperfused “defect” regions, with studies using varying thresholds determined through Otsu’s algorithm,^90^ k-means clusters,^88^ or a percentile of the signal from healthy parenchymal tissue.^89^

#### DCE-MRI perfusion applications in COPD

**Figure 5.**
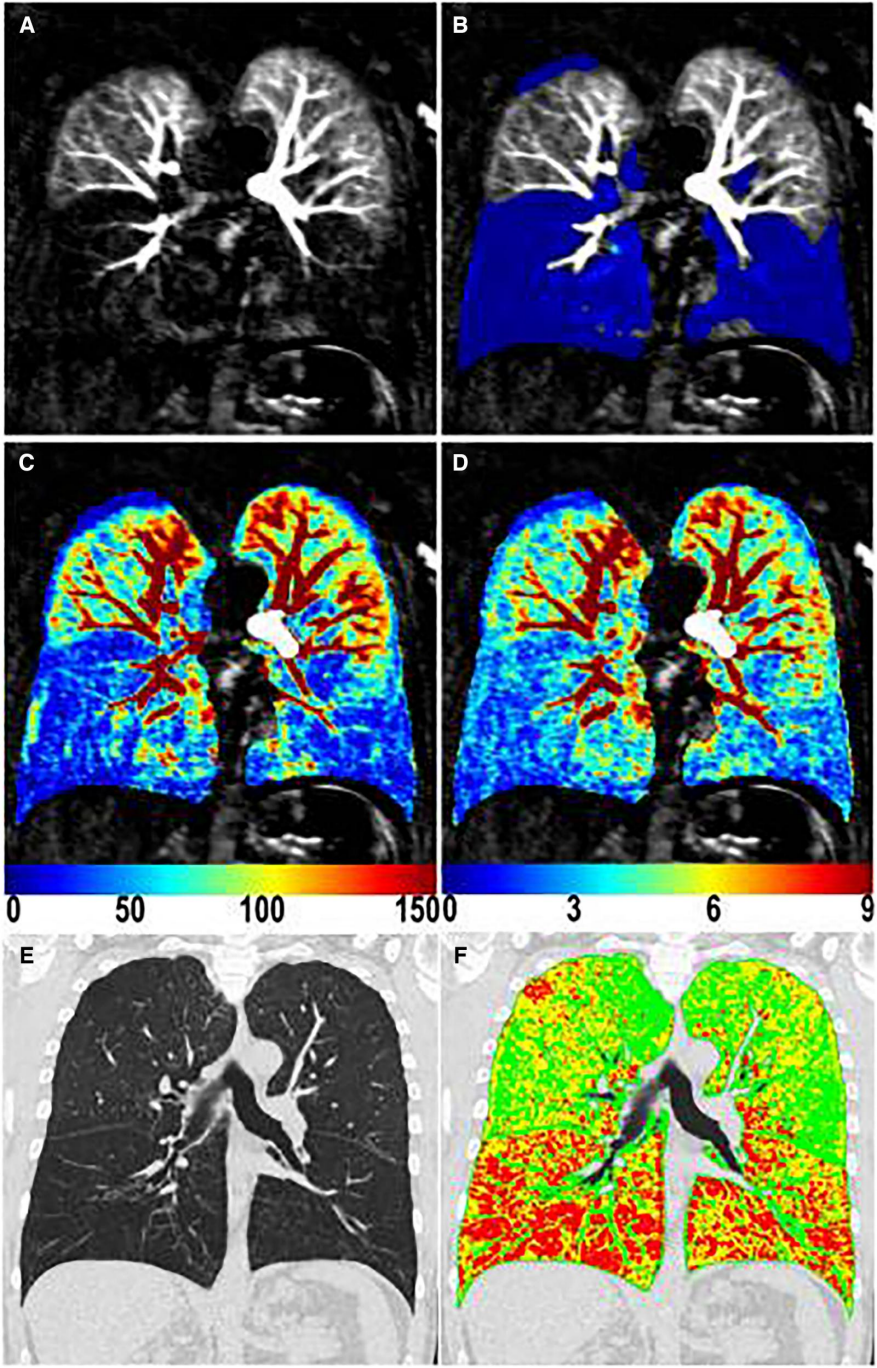
Representative DCE-MRI and CT of a 51-year-old female patient with COPD GOLD 2 with FEV1%predicted = 53.26%, FEV1/FVC = 0.53, and MRI perfusion score = 10. (A) Residue function map at the time point of maximum contrast enhancement (Rmax map), (B) corresponding map of perfusion defects in percent (QDP map, blue) calculated with Otsu’s method (QDP = 72.94%), (C) corresponding map of the pulmonary blood flow (PBF = 30.19 mL/100 mL/min), (D) corresponding map of the pulmonary blood volume (PBV = 2.34 mL/100 mL), (E) coronal CT, and (F) corresponding parametric response map (PRM map) are presented. PRM classifies the voxels of the lung into normal lung tissue (28.23%, green), functional small airway disease (fSAD = 34.96%, yellow), and emphysema (36.13%, red). Reproduced with permission from Schiwek et al, Eur Radiol, 2022; 32, 1879-1890. © 2022 Springer Nature.^88^

Repeatability of DCE-MRI quantified QDP measurements taken 1-month apart in those with stable COPD has been poor, with significant differences being shown^90^; reproducibility analysis of these measurements between scans obtained at varying echo times in COPD have also been shown to exhibit a TE-dependence.^91^ However, good agreement between DCE-MRI QDP and radiologist visual scoring of perfusion defects in COPD has been demonstrated.^88^ Significant associations have also been reported between DCE-MRI QDP with measurements of FEV_1_ and FEV_1_/FVC and CT quantified fSAD and emphysema, as shown in Figure 5.^88^ Yet, associations with other important cross-sectional outcomes like symptoms, exercise capacity, and longitudinal outcomes needs to be investigated. Importantly, longitudinal DCE-MRI QDP measurements post-administration of IND/GLY-based bronchodilators have shown decreases in functional gas-exchange impairments in response to treatment.^92^

#### ^1^H-PREFUL-MRI perfusion applications in COPD

^1^H-PREFUL-MRI analysis to generate perfusion weighted maps has recently gained popularity as a safe, contrast-free alternative imaging technique. Multiple published studies including a meta-review have investigated the agreement between DCE-MRI QDP and ^1^H-PREFUL-MRI quantified QDP, showing good concordance and moderately strong correlations between the 2 imaging techniques.^25^^,^^77^^,^^89^ Unlike DCE-MRI QDP, ^1^H-PREFUL-MRI QDP has shown excellent repeatability in those with COPD,^79^ but reproducibility across different scanner parameters, such as field strength in healthy volunteers has been poor,^81^ and further investigation is warranted for determining the optimal scanner settings. Increased ^1^H-PREFUL-MRI QDP have been demonstrated in those along the COPD continuum^89^ and with pulmonary hypertension.^93^ In COPD, associations for ^1^H-PREFUL-MRI QDP with FEV_1_^76^,^77^,^89^ have been shown, but associations with symptoms, exercise capacity, or longitudinal outcomes have not been investigated. Finally, ^1^H-PREFUL-MRI QDP has also demonstrated the ability to quantify gas-exchange changes in response to IND/GLY-based bronchodilators.^94^

#### HP-MRI gas exchange in COPD

Uniquely, HP-MRI enables imaging of diffusion directly in the alveolar-capillary (barrier) and red blood cell (RBC) compartments individually, as it has a distinct resonance in each diffusive phase that can be measured.^95^ The RBC to barrier ratio (RBC: barrier) has provided insights into abnormalities occurring in the pulmonary capillaries and can reflect impaired gas transfer.^96^ Individuals with COPD who have preserved RBC: barrier measurements prior to treatment with bronchodilators exhibit greater improvements in ventilation post-treatment, indicating that evaluation of perfusion abnormalities before therapeutic treatment could be an important consideration for clinicians.^60^ Diffusion-weighted HP-MRI (DW-HP-MRI) is another imaging technique leveraging HP gases that can probe alveolar size through a measurement known as the apparent diffusion coefficient (ADC). Higher ADC values (measured in units of cm^2^/s) represent faster motion of the inhaled HP gas within the alveolar space, and therefore indirectly describes the increased breakdown of the alveolar membrane. This hypothesis is further supported by multiple studies showing increased ADC measurements in those with COPD relative to healthy controls,^54^ and significant associations with diffusion capacity of the lungs for carbon monoxide (DLCO) measurements as well as CT-measured emphysema.^97^

#### Additional perfusion biomarkers

Additional qMR imaging biomarkers have been developed and implemented in COPD to quantify gas-exchange. The direct rate of pulmonary blood flow (PBF) in the lungs can be obtained using DCE-MRI or ^1^H-PREFUL-MRI (alternatively termed Q).^25^ Reduced PBF has been shown in individuals with COPD, including mild COPD individuals without emphysema.^87^ Although coupled reductions in both ventilation and perfusion typically occur due to hypoxic pulmonary vasoconstriction, gas-exchange can sometimes be independently reduced without ventilatory impairment due to conditions such as acute pulmonary embolisms, an important comorbidity in COPD.^98^ Evaluating ventilation-perfusion mismatch (and by-extension mismatch), can be performed using a qMR biomarker known as VQM, defined as regions on VDP and QDP masks that are classified as either both a defect or both healthy tissue.^94^ Improvements in spatial-matching of ventilation and perfusion after administration of a bronchodilator, quantified using changes in VQM, has been demonstrated.^94^

## Gaps in the literature

The rapid technical development of lung MRI techniques and qMR biomarkers that allow for the characterization of structural and functional lung disease manifestations has been tremendous, yet, further technical development and clinical validation is required. A summary of the current state-of-the-art progress in technical developments for qMR imaging biomarkers, including repeatability, reproducibility, and associations with gold standard CT measurements is shown in Table 2. First, there have been deep-learning networks developed to automate biomarker extraction pipelines, but open-source models are currently limited and strictly focus on the lung parenchyma, and not the airways or vasculature.^33^^,^^35^ Creating such accessible and fully-automated pipelines will accelerate qMR imaging biomarker research. The performance of these models will also need to be evaluated irrespective of field strength, pulse sequence, echo-time, and imaging centre to ensure interscan and intersite reproducibility and limit potential sources of variability. Furthermore, as most development cohorts have seen limited inclusion of COPD participants, future work should look to either re-train disease-specific deep-learning models for, or externally validate their performance in COPD. Although these differences are inherently important in evaluating the generalizability of deep-learning models, their impact may also have broader clinical implications for simpler image quality metrics such as the signal-noise-ratio (SNR) and contrast-noise-ratio (CNR). Differences across k-space trajectories, scanner calibrations, and reconstruction algorithms between scanner manufacturers (GE vs. Siemens vs. Philips) should also be accounted for when extracting qMR imaging biomarkers.

**Table 2.** Overview of the state-of-the-art progress in technical developments of qMR imaging biomarkers.

Imaging biomarkers	Intersession repeatability	Interscan reproducibility	Validation against histology	Associations with visual scoring	Associations with CT/SPECT
Structural biomarkers					
Emphysema	**✓** ^37^	**-**	**-**	**-**	**✓** ^27^
Airway morphology	**-**	**-**	**-**	**-**	**-**
Ventilation biomarkers					
HP-MRI VDP	**✓** ^68^	**✓** ^69^	–	**✓** ^113^	**✓** ^58^
^19^F-MRI VDP	**✓** ^73^	–	–	–	–
^1^H-PREFUL-MRI VDP	**✓** ^79^	**✓** ^81^	–	**✓** ^114^	**✓** ^115^
Perfusion biomarkers					
DCE-MRI QDP	**✓** ^90^	**✓** ^91^	–	**✓** ^88^	**✓** ^25^
^1^H-PREFUL-MRI QDP	**✓** ^79^	**✓** ^81^	–	**✓** ^116^	**✓** ^25^
HP-MRI RBC: barrier	**✓** ^117^	**✓** ^118^	–	**✓** ^119^	**✓** ^120^

Abbreviations: COPD = chronic obstructive pulmonary disease; CT = computed tomography; DCE = dynamic contrast enhanced; HP: hyperpolarized; N/A: not applicable; PREFUL: phase-resolved functional lung; qMR = quantitative magnetic resonance; RBC: red blood cell; SPECT = single positron emission computed tomography.

Although functional qMR imaging biomarkers describing ventilation and perfusion in COPD have been established, studies focusing on the development and validation of structural qMR imaging biomarkers for application in those with COPD are limited; a complete summary of what has been demonstrated by each major qMR biomarker in the literature for cross-sectional analyses, longitudinal analyses, and acute response to therapy is shown in Table 3. For example, histopathological validation of the ideal signal intensity threshold for quantifying emphysema and quantitative evaluation of wall-thickening on MRI using established CT-metrics like wall area % (WA%) should be performed. Comparison of these biomarkers between qMR and the gold-standard lung qCT biomarkers in the same participants must also be demonstrated. Large, multicentre, longitudinal, observational COPD cohorts with a direct focus on developing and investigating qMR imaging biomarkers characterizing structural and functional abnormalities are also needed. In addition, studies have leveraged lung CT radiomics for COPD applications, including predicting cross-sectional outcomes like COPD status and severity,^99–101^ and longitudinal outcomes like lung function decline,^102^ incident COPD,^103^ and mortality.^55^ However, investigations for application of MRI radiomics in COPD are limited,^104^^,^^105^ and no studies have investigated the repeatability/reproducibility of these measurements or have used large, multicentre cohorts.

**Table 3.** Overview of the state-of-the-art progress in determining associations for qMR imaging biomarkers with key clinical outcomes in individuals with COPD.

Imaging biomarkers	Discrimination of disease	Associations with cross-sectional outcomes			Associations with longitudinal outcomes			Sensitivity to treatment response
		Lung function	Symptom scores	Exercise capacity	Lung function decline	Future exacerbations or hospitalizations	Mortality	
Structural biomarkers								
Emphysema	**✓** ^27^	**✓** ^27^	**-**	**-**	**-**	**-**	**-**	**-**
Airway morphology	**-**	**-**	**-**	**-**	**-**	**-**	**-**	**-**
Ventilation biomarkers								
HP-MRI VDP	**✓** ^76^	**✓** ^54^	**✓** ^57^	**✓** ^70^	**✓** ^57^	**✓** ^53^	**✓** ^55^	**✓** ^60^
^19^F-MRI VDP	**✓** ^74^	**✓** ^61^	–	–	–	–	–	**✓** ^74^
^1^H-PREFUL-MRI VDP	**✓** ^78^	**✓** ^75^	–	–	–	–	–	**✓** ^83^
Perfusion biomarkers								
DCE-MRI QDP	**✓** ^88^	**✓** ^88^	–	–	–	–	–	**✓** ^92^
^1^H-PREFUL-MRI QDP	**✓** ^89^	**✓** ^89^	–	–	–	–	–	**✓** ^94^
HP-MRI RBC: barrier	**✓** ^121^	**✓** ^122^						**✓** ^60^

Abbreviations: COPD = chronic obstructive pulmonary disease; DCE = dynamic contrast enhanced; HP: hyperpolarized; PREFUL: phase-resolved functional lung; qMR = quantitative magnetic resonance; RBC: red blood cell.

For future widespread use of structural and functional qMR imaging biomarkers, a standardized approach to quantification is required, similar to the one proposed by the Quantitative Imaging Biomarker Alliance (QIBA) for measurements of lung density on CT.^106^ For example, questions like: (1) which functional defect identification pipeline and structural biomarkers are invariant to scan changes, and are repeatable, reproducible, and correlate best with outcomes-of-interest; and (2) what are their minimal clinically important differences, need to be answered. Such advances can tremendously benefit clinical care of COPD patients as research into therapies to slow disease progression will be supported by morpho-functional (structural and functional) quantitative biomarkers that can be safely extracted without radiation, making them suitable as endpoints to guide and monitor treatment response.

Finally, it is also important to note the recent advancements in low-field MRI (<1 T) for applications in lung imaging.^107–109^ Low-field MRI has distinct advantages such as an improved field homogeneity and enhanced NMR relaxation properties which reduces air-tissue interface distortions and is particularly useful for accurate measurements of potential microstructural lung changes due to emphysema, inflammation, and ventilation-perfusion mismatch captured through shortened T1 times and prolonged T2* times.^109^^,^^110^ Such developments have also expanded the potential clinical utility and accessibility of MRI, allowing for the creation of imaging systems that are smaller, have an open-design, cost less, and can even image some patients with metallic implants. However, low-field MRI technology is still a developing novel technology, and more studies are required to compare its COPD-specific diagnostic capabilities to the more established 1.5 T MRI, 3 T MRI, and CT modalities. Furthermore, enhanced detection of changes in T1 and T2* times can be further leveraged using oxygen-enhanced MRI, which has shown some potential to detect ventilation abnormalities and compliment CT biomarkers in smokers and those early-COPD, but more work is required to validate its efficacy using low-field MRI.^111^^,^^112^

## Conclusions

Lung imaging using novel MRI techniques now allows for the characterization of structure and function to obtain quantitative information describing the parenchyma, airways, ventilation, and perfusion in those with COPD without ionizing radiation. More specifically, UTE-^1^H-MRI can produce CT-like structural images of the lungs at submillimetre resolution, while intravenous- and inhalation-based contrast techniques like DCE-MRI, HP-MRI, and ^19^F-MRI create functional maps of air-flow and blood-flow. Further, advances in respiratory-motion tracking and time-wise analysis techniques like ^1^H-PREFUL-MRI allow for morpho-functional imaging to be obtained in a single scanning session during free-breathing. Quantitative MR imaging biomarkers extracted from the aforementioned imaging techniques have demonstrated associations with important cross-sectional COPD outcomes like lung function, and have been sensitive to bronchodilators. However, further studies are required to demonstrate concordance between qMR biomarkers with gold standard qCT biomarkers, particularly for the airways, establish standardized extraction protocols that have evaluated important technical considerations like repeatability and reproducibility, and evaluate associations to other important cross-sectional and longitudinal COPD outcomes. Such efforts will help establish the role of MRI for routine long-term clinical care and help improve patient outcomes in patients with COPD.
